# Artificial Intelligence in Health Promotion and Disease Reduction: Rapid Review

**DOI:** 10.2196/70381

**Published:** 2025-08-01

**Authors:** Farzaneh Yousefi, Florian Naye, Steven Ouellet, Achille-Roghemrazangba Yameogo, Maxime Sasseville, Frédéric Bergeron, Marianne Ozkan, Martin Cousineau, Samira Amil, Caroline Rhéaume, Marie-Pierre Gagnon

**Affiliations:** 1 Department of Health Management, Policy, and Economics, Faculty of Management and Medical Information Sciences, Kerman University of Medical Sciences, Kerman, Iran; 2 Faculty of Nursing Sciences, Université Laval, 1050, avenue de la Médecine, Québec, QC, G1V 0A6, Canada, 1 418 656 2131 ext 407576; 3 Faculty of Medicine and Health Sciences, School of Rehabilitation, Université de Sherbrooke, Sherbrooke, QC, Canada; 4 VITAM Research Center on Sustainable Health, Québec, QC, Canada; 5 The International Observatory on the Societal Impacts of AI and Digital Technologies, Québec, QC, Canada; 6 Library, Université Laval, Québec, QC, Canada; 7 Faculty of Law, University of Ottawa, Ottawa, ON, Canada; 8 Department of Logistics and Operations Management, HEC Montréal, Montreal, QC, Canada; 9 School of Nutrition, Université Laval, Québec, QC, Canada; 10 Department of Family Medicine and Emergency Medicine, Faculty of Medicine, Université Laval, Québec, QC, Canada; 11 Research Center of Quebec Heart and Lungs Institute, Quebec, QC, Canada

**Keywords:** artificial intelligence, health promotion, disease reduction, AI in health, rapid review, SWOT analysis

## Abstract

**Background:**

Chronic diseases represent a significant global burden of mortality, exacerbated by behavioral risk factors. Artificial intelligence (AI) has transformed health promotion and disease reduction through improved early detection, encouraging healthy lifestyle modifications, and mitigating the economic strain on health systems.

**Objective:**

The aim of this study is to investigate how AI contributes to health promotion and disease reduction among Organization for Economic Co-operation and Development countries.

**Methods:**

We conducted a rapid review of the literature to identify the latest evidence on how AI is used in health promotion and disease reduction. We applied comprehensive search strategies formulated for MEDLINE (OVID) and CINAHL to locate studies published between 2019 and 2024. A pair of reviewers independently applied the inclusion and exclusion criteria to screen the titles and abstracts, assess the full texts, and extract the data. We synthesized extracted data from the study characteristics, intervention characteristics, and intervention purpose using structured narrative summaries of main themes, giving a portrait of the current scope of available AI initiatives used in promoting healthy activities and preventing disease.

**Results:**

We included 22 studies in this review (out of 3442 publications screened), most of which were conducted in the United States (10/22, 45%) and focused on health promotion by targeting lifestyle dimensions, such as dietary behavior (10/22, 45%), smoking cessation (6/22, 27%), physical activity (4/22, 18%), and mental health (3/22, 14%). Three studies targeted disease reduction related to metabolic health (eg, obesity, diabetes, hypertension). Most AI initiatives were AI-powered mobile apps. Overall, positive results were reported for process outcomes (eg, acceptability, engagement), cognitive and behavioral outcomes (eg, confidence, step count), and health outcomes (eg, glycemia, blood pressure). We categorized the challenges, benefits, and suggestions identified in the studies using a Strengths, Weaknesses, Opportunities, and Threats analysis to inform future developments. Key recommendations include conducting further investigations, taking into account the needs of end users, improving the technical aspect of the technology, and allocating resources.

**Conclusions:**

These findings offer critical insights into the effective implementation of AI for health promotion and disease prevention, potentially guiding policymakers and health care practitioners in optimizing the use of AI technologies in supporting health promotion and disease reduction.

## Introduction

As per the World Health Organization, noncommunicable diseases are responsible for 41 million deaths annually, constituting 71% of the total mortality [1-3]. These diseases are characterized by prolonged duration, risks of complications, and long-term treatment, significantly impacting people’s health and quality of life [4]. Behavioral risk factors such as smoking, inadequate nutrition, alcohol consumption, and lack of physical activity [5] contribute to the development of these conditions [6]. Chronic diseases have emerged as a significant challenge to global health, which underscores the crucial need for a targeted approach in health policy to address this issue [6,7]. Actively promoting the prevention and management of chronic diseases is essential to achieving broader health care goals, such as (1) improving the patient experience, (2) improving population health, (3) reducing costs, (4) promoting equity, (5) supporting health care providers, and (6) enhancing system efficiency, referred to as the “sextuple aim” [8].

Most chronic diseases can be prevented or improved through health promotion strategies [9], thereby mitigating risk factors through the dissemination of lifestyle information and advocacy of healthy habits [10]. Successful health promotion programs are those that focus on imparting knowledge, influencing attitudes, and changing behaviors [11]. Health promotion strategies, such as enhancing public awareness, changing behavior, creating supportive environments, and promoting healthy eating and active lifestyles, are key to preventing chronic diseases [12,13]. In this context, self-management is a crucial approach to managing chronic conditions, emphasizing patient responsibility and problem-solving [14,15]. Health promotion refers to proactive efforts aimed at empowering individuals and communities to adopt healthier lifestyles, prevent diseases, and enhance overall well-being [16,17]. Disease reduction, on the other hand, focuses on minimizing the incidence, severity, and long-term impacts of chronic conditions through targeted interventions and preventive strategies [18].

Recent advancements in artificial intelligence (AI) can be widely used in the medical field to improve patient care [19] and can also be effective in encouraging healthy behaviors and lifestyle changes [20]. AI, as a branch of computer science, simulates human cognition to perform tasks such as reasoning, learning, and decision-making [21,22]. Its core techniques, such as machine learning, deep learning, natural language processing, knowledge representation, and reasoning, have been deployed in health research areas [23], increasing its popularity in the medical community [22-25]. It has offered significant potential for public health practitioners and policymakers by supporting context-specific, data-driven interventions [26]. AI’s ability to reason, act, and adapt based on data is particularly valuable in the early prevention of diseases [27], treatment optimization [24], and delivering personalized, engaging self-care solutions [27,28].

The positive influence of AI in health care can include significant transformations in health promotion and disease prevention. Early disease detection and prevention can motivate the population to adopt healthy eating, lifestyle, and exercise habits [29]; improve patient outcomes by lowering disease severity; and reduce the economic burden on health systems [30]. However, the acceptance and application of these technologies for health, especially in certain populations such as older adults, are much slower than their emergence and development [31]. This highlights the importance of identifying practical applications of AI in promoting healthy lifestyles to reduce the risk of diseases, as well as the essential conditions for its effective implementation.

Evidence of AI and machine learning applications for health promotion has been synthesized specifically for promoting health behavior change, physical activity, and healthy eating [20,32,33]. However, most existing research has primarily focused on AI applications in clinical settings and individualized interventions, with limited attention to its role in community-based and nonclinical health promotion programs. AI-driven technologies, such as digital health assistants, mobile interventions, and community engagement platforms, have the potential to enhance public health initiatives by increasing the accessibility, scalability, and personalization of preventive strategies [34,35]. Despite this potential, there remains a lack of synthesized knowledge on the implementation of AI in broader, nonclinical contexts, which limits its integration into public health policies and community-wide interventions. This gap in evidence further restricts the ability to apply AI-based solutions effectively in real-world health promotion and disease prevention strategies.

This review thus aims to: (1) identify AI-based initiatives and their purposes; (2) synthesize their impacts on health promotion domains; and (3) categorize the challenges, benefits, and suggestions regarding successful AI implementation in health promotion and disease reduction, using a Strengths, Weaknesses, Opportunities, and Threats (SWOT) matrix [36].

## Methods

### Overview

We conducted a rapid knowledge synthesis following the Cochrane Collaboration guidance for rapid reviews [37]. The results are presented based on the PRISMA (Preferred Reporting Items for Systematic Reviews and Meta-Analyses) guidelines [38,39]. We prospectively registered the protocol for this rapid review in the Open Science Framework Registries [40].

### Eligibility Criteria

Our search strategy was guided by the Population, Concept, and Context framework, recommended by the Johanna Briggs Institute [41,42], to ensure a comprehensive and focused search. This framework suited our study due to its appropriateness for a broad research subject (Table 1).

**Table 1.** The Population, Concept, and Context framework – inclusion and exclusion criteria.

Element	Inclusion criteria	Exclusion criteria
Population	Any population or community in OECD^a^ countries that has health promotion policies to prevent or reduce chronic diseases, without restrictions on age or other demographic characteristics. The list of OECD countries is available in Multimedia Appendix 1.	Focus on a narrow population (eg, a company, a hospital, a school)
Concept	Studies about AI^b^-based initiatives aimed at health promotion including healthy lifestyle, physical activity, healthy nutrition and diet, healthy behavior, stress management, smoking and alcohol reduction, health education, healthy activities engagement, and health. Studies with an AI intervention, AI-based techniques, and AI algorithms for preventing diseases at primary, secondary, and tertiary levels. Health promotion in this review refers to AI-driven interventions designed to support behavior change, encourage preventive health measures, and enhance self-management of chronic conditions through digital tools such as mobile apps, chatbots, and personalized health interventions.	Using AI for epidemiological, surveillance, administrative purposes, or operational aspects by health care providers. Intervention just focuses on chronic disease management, not health promotion. Studies with methods or interventions not directly related to AI (eg, Internet of Things, robotics).
Context	Focus on OECD countries, including interventions at the level of cities, provinces, or countries. Encompass a broad range of cultural and geographical settings, reflecting the diverse applications of AI in health care across different contexts.	Algorithms used by health care providers for support in administrative tasks and for operational aspects or clinical decisions (eg, diagnostic, treatment)

a OECD: Organisation for Economic Co-operation and Development.

b AI: artificial intelligence.

### Literature Search

We developed a comprehensive list of keywords, synonyms, and MeSH (Medical Subject Headings) terms related to the main concepts of our research question, guided by the Population, Concept, and Context framework. This strategy was formulated in collaboration with an information specialist (FB), aiming to capture the broad spectrum of AI applications in health promotion and disease prevention. We searched two scientific databases aligned with the subject of interest, MEDLINE (Ovid) and CINAHL, in March 2024, to locate studies published between January 1, 2019, and March 15, 2024 (Multimedia Appendix 2). No other restrictions were applied. Since the use of AI in health has been promoted in recent years, focusing on the literature of the last 5 years provides us with more up-to-date findings, as this field evolves rapidly.

### Study Selection, Data Extraction, and Synthesis

We exported all citations to the Covidence web-based collaborative tool [43], where duplicates were removed manually and using the automated function. Seven reviewers (FY, FN, SO, MPG, MC, MO, MS) working in pairs performed an independent assessment of titles and abstracts, and then 5 (FY, FN, MPG, SO, ARY) reviewed full texts. Any discrepancies between reviewers during the title and abstract screening or full-text review were resolved through discussion. If consensus could not be reached, a senior reviewer (MPG) made the final decision. We performed a calibration exercise on 10 citations to ensure that the selection criteria were understood in the same way by all reviewers. The same reviewers completed data extraction in an Excel 2016 (Microsoft Corp) form, including study characteristics (eg, authors, title, year, country, design, and population data), intervention characteristics (type of AI initiatives), intervention purpose (eg, health promotion domain, impacts), and AI implementation (challenges, opportunities, and suggestions). A single reviewer extracted data, which was then confirmed by a senior reviewer (MPG). In cases where discrepancies arose during data extraction, they were discussed and resolved collectively. When needed, the senior reviewer (MPG) provided the final decision to ensure consistency in data reporting. Although initial data extraction was conducted by a single reviewer, all extracted data were independently verified by a senior reviewer to minimize bias and enhance reliability. This two-step verification approach aligns with established practices in rapid reviews [44]. We used a PRISMA 2020 flow diagram [45] to describe the study identification, screening process, and application of inclusion and exclusion criteria.

We synthesized the extracted data from study characteristics, intervention characteristics, and intervention purpose using structured narrative summaries of main themes, which gave a portrait of the current scope of available AI initiatives used in promoting healthy activities and preventing disease. We grouped the reported outcomes into three categories, namely, knowledge-cognition-behavior, health status, and process. We used thematic analysis [46] to categorize and summarize the challenges, benefits, and suggestions related to AI implementation in health promotion and disease prevention, according to a SWOT matrix.

**Figure 1.**
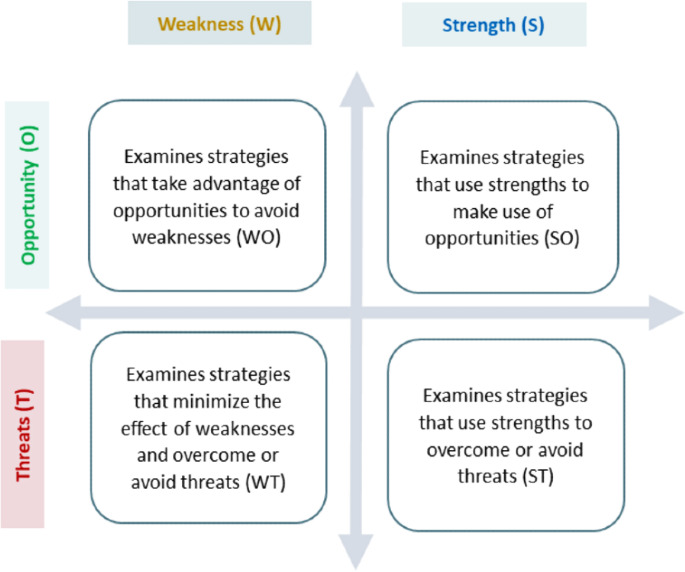
SWOT matrix and its strategies. O: Opportunity; S: Strength; T; Threat; W: Weakness.

A SWOT analysis systematically evaluates an organization’s internal strengths and weaknesses alongside external opportunities and threats within its environment. This internal analysis aims to identify the organization’s resources, capabilities, core competencies, and competitive advantages, categorized as Strengths and Weaknesses. Conversely, the external analysis assesses market opportunities and threats by examining competitor resources, the industry landscape, and broader environmental factors grouped as Opportunities and Threats. The goal of a SWOT analysis is to leverage insights about the organization’s internal and external contexts to inform strategic decision-making [36]. This matrix identifies potential tactical strategies for exploiting opportunities or mitigating threats by leveraging existing strengths and minimizing weaknesses, facilitating the development of tactical strategies from 4 distinct perspectives [36,47,48] (Figure 1).

## Results

### Study Selection

**Figure 2.**
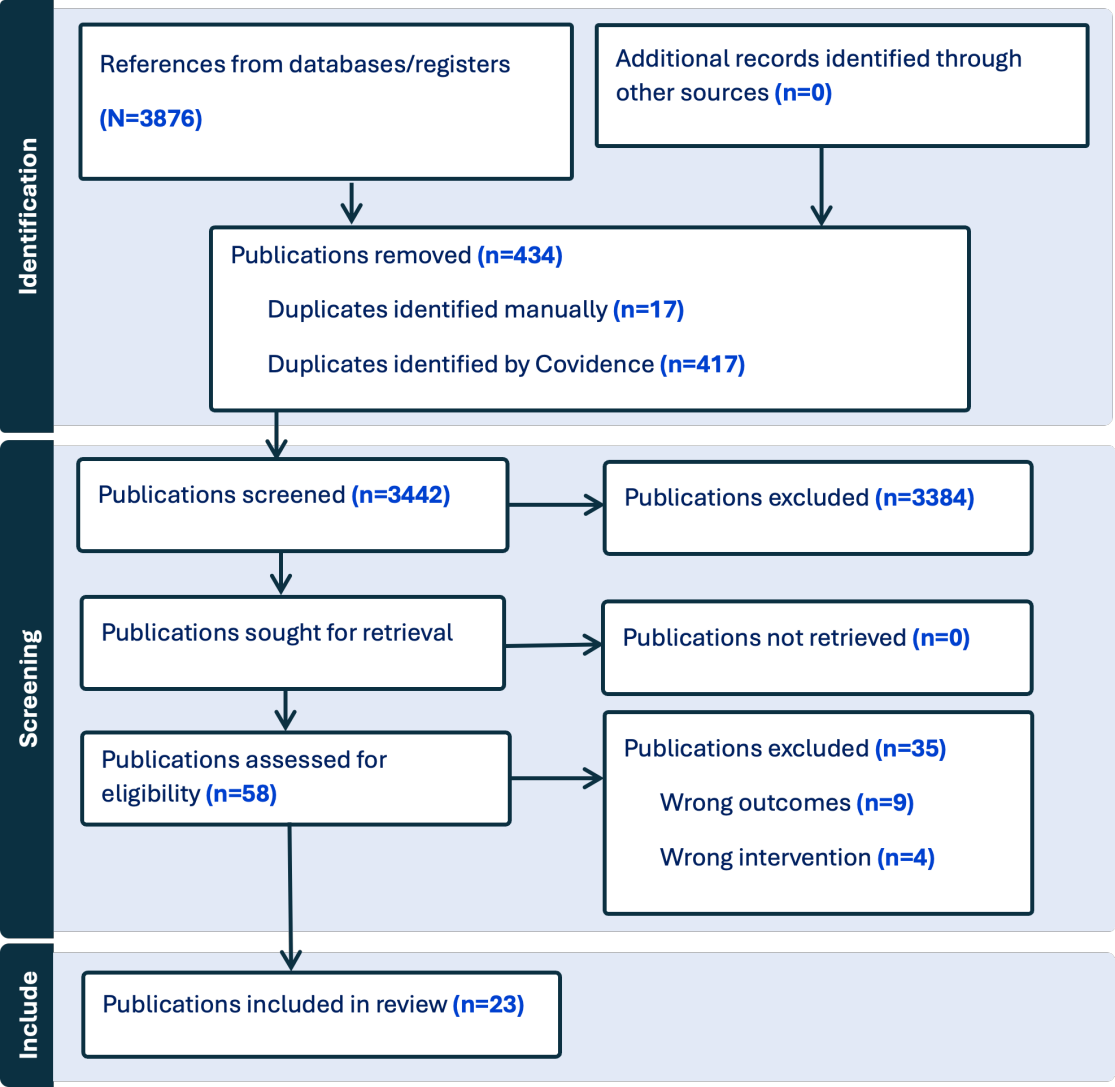
PRISMA flow diagram of the study inclusion process. PRISMA: Preferred Reporting Items for Systematic Reviews and Meta-Analyses.

A total of 3876 publications were retrieved, and 434 duplicates were removed both manually and automatically (by Covidence). The remaining 3442 publications were screened by independent reviewers using titles and abstracts. Among them, 58 publications were screened in full text, resulting in 23 publications describing 22 studies suitable for inclusion in this review (see Figure 2).

### Characteristics of the Included Studies

**Figure 3.**
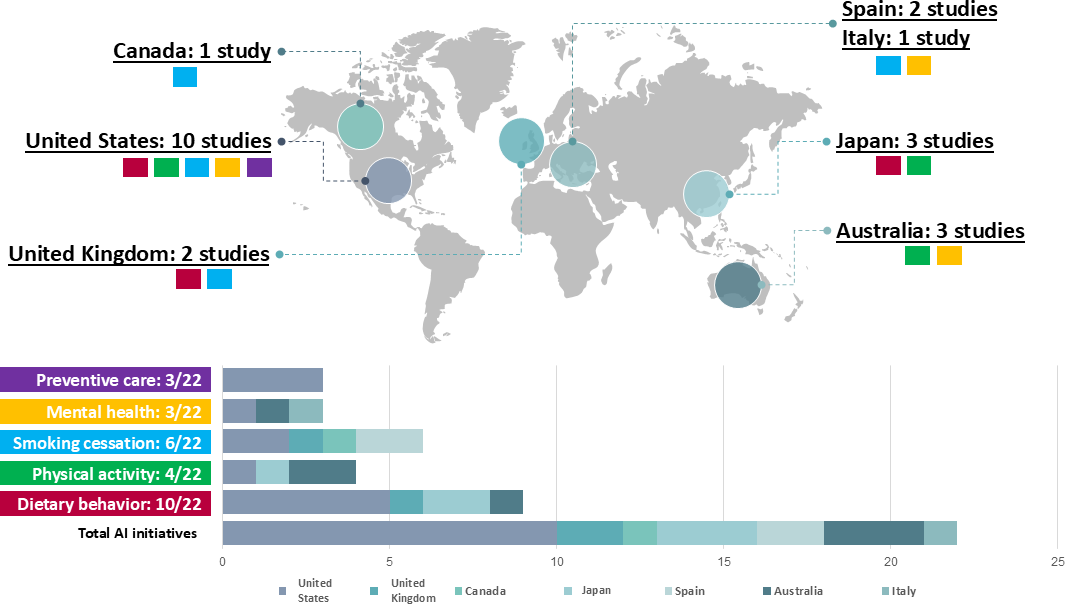
The geographical distribution of studies and their focus on different health promotion domains. AI: artificial intelligence.

The included studies were published between 2019 and 2023 (see Table 2 and Figure 2), and most of them (7/22, 32%) were published in 2023. Ten studies (45.5%) were conducted in the United States, 3 (14%) in Japan, 3 (14%) in Australia, 2 (9%) in the United Kingdom, 2 (9%) in Spain, 1 (4%) in Canada, and 1 (4%) in Italy (see Figure 3). The higher representation of studies from the United States may be attributed to greater investment in AI research, a well-established digital health infrastructure, and large-scale government-supported AI initiatives, facilitating AI-driven public health applications [49,50]. The number of participants involved in each study ranged from 21 to 139,164, with participants generally being recruited voluntarily and without any specific health concerns. Of the 22 studies included in our rapid review, 11 (50%) were randomized or nonrandomized trials, 3 (14%) were cohort studies, 2 (9%) were feasibility studies, 2 (9%) were observational studies, 1 (4%) study used a mixed method research design, and 1 (4%) study used a quasi-experimental design (see Table 2).

**Table 2.** Characteristics of the included studies.

Studies	Country	Year	Aim of the study	Study design	Study participants	Type of AI^a^ systems	Intervention purpose
Amiri et al, 2023 [51]	United States	2023	To create an AI-powered meal planner that generates personalized healthy meal plans based on the user’s health conditions, preferences, and status	Feasibility study	39	Mobile app: meal planning system	Dietary behavior
Branch et al, 2022 [52]	United States	2022	To evaluate changes in blood pressure and body weight after participating in the fully digital, AI-powered Lark Hypertension Care program.	Observational study	864	Mobile app: Lark Hypertension Care	Dietary behavior, preventive care (hypertension)
Brinsley et al, 2023 [53]	Australia	2023	To demonstrate proof-of-concept for a chatbot-led digital lifestyle medicine program in aiding rehabilitation for return to work.	Retrospective cohort study	78 adults	Software/website: Lucy (virtual health assistant)	Mental health
Brown et al, 2023 [54]	Canada	2023	To design, evolve, and measure the effectiveness of a chatbot system that can guide ambivalent people who smoke toward the decision to quit smoking with motivational interviewing–style generative reflections.	Clinical trial	349 current smokers	Software/website/ chatbot: MIBot, 4 versions	Smoking cessation
Bucher et al, 2022 [55]	United States	2022	To establish the feasibility of a reinforcement learning–enabled mammography digital health intervention delivered via email, including understanding its reach and ability to elicit behavioral outcomes related to scheduling and attending mammograms among women of different ages, races, educational attainment levels, and household incomes.	Observational study	139,164 women eligible for mammograms	Other: HealthOrg (precision nudging for mammography)	Preventive care (mammogram)
Bul et al, 2023 [56]	United Kingdom	2023	To assess the usability and initial effectiveness of a web-based AI-driven nutrition platform designed to assist individuals with diabetes and their caregivers in identifying healthy recipes, meal planning, and online shopping.	Mixed methods study	73 adults with type 1 or type 2 diabetes	Website/platform: AI-driven web-based nutrition platform	Dietary behavior
Carrasco-Hernandez et al, 2020 [57]	Spain	2020	To analyze the long-term efficacy of a mobile app supporting psychopharmacological therapy for smoking cessation and complementarily assess the involved innovative technology.	Randomized controlled trial	196 smokers	Mobile app: N/A	Smoking cessation
Chen et al, 2021 [58]	United States	2021	To assess user engagement with a recommender system that actively sought feedback on each message to improve message selection for promoting smoking cessation and examine the impact of this engagement on cessation outcomes.	Prospective cohort study	731 current smokers	Other: computer-tailored health communication systems	Smoking cessation
Danieli et al, 2021 [59]	Italy	2021	To evaluate a protocol for an intervention that integrates a conversational AI-powered mobile app with traditional psychotherapy for treating work-related stress and anxiety.	Randomized trial	21	Mobile app: therapy empowerment opportunity (TEO)	Mental health
Faro et al, 2023 [60]	United States	2023	To compare a machine learning recommender (ML recommender) for tailoring motivational text messages versus a standard text-based motivational intervention (standard messaging) and to evaluate the impact of a viral peer-recruitment tool kit (viral tool kit) versus no tool kit in a smoking-cessation intervention.	Randomized clinical trial	1487	Website: tool kit (computer-tailored health communication systems)	Smoking cessation
Forman et al, 2019 [61]	United States	2019	To evaluate the feasibility, acceptability, and preliminary effectiveness of OnTrack among weight loss program participants	Open trial design	44 people with overweight or obesity	Mobile app: OnTrack	Dietary behavior
Graham et al, 2022 [62]	United States	2022	To evaluate an alternative delivery method of the National Diabetes Prevention Program (DPP) powered by a conversational AI called Lark DPP.	Retrospective, longitudinal cohort study	191	Mobile app: Lark DPP AI-powered coaching	Dietary behavior
Hassoon et al, 2021 [63]	United States	2021	To determine if novel AI coaching interventions increase physical activity among overweight or obese and physically inactive cancer survivors compared to a control group receiving standard health information.	Randomized trial	42	Mobile app: MyCoach and SmartText	Physical activity
Maher et al, 2020 [64] and Davis et al, 2020 [65]	Australia	2020	To test the feasibility (recruitment and retention) and preliminary efficacy of physical activity and a Mediterranean-style dietary intervention (MedLiPal) delivered via an AI virtual health coach.	Nonrandomized Trial	31	Mobile app/website: Paola	Physical activity, dietary behavior
Nakata et al, 2022 [66]	Japan	2022	To test the hypothesis that CALO Mama Plus could promote body weight reduction in Japanese adults with overweight or obesity.	Randomized controlled trial	141 people with overweight or obesity	Mobile app: CALO Mama Plus	Dietary behavior
Okaniwa et al, 2022 [67]	Japan	2022	To investigate whether AI alone can effectively encourage healthy behaviors or whether human interventions are needed to achieve and sustain health-related behavior change.	Nonrandomized controlled trial	102	Mobile app developed by Asken Inc	Dietary behavior
Olano-Espinosa et al, 2022 [68]	Spain	2022	To assess the effectiveness of a chatbot-based intervention for quitting smoking via smartphones compared to usual clinical practice in primary care.	Randomized clinical trial	513	Mobile app/chatbot: Dejal@bot	Smoking cessation
Perski et al, 2019 [69]	United Kingdom	2019	To assess whether the version of the Smoke-Free app with a supportive AI-powered chatbot leads to increased engagement and short-term quit success compared to a version without the chatbot.	Randomized trial	57,214 adult smokers	Chatbot: Smoke-Free app	Smoking cessation
Stephens et al, 2019 [70]	United States	2019	To assess the feasibility of integrating Tess, an AI chatbot, into behavioral counseling for adolescent patients managing weight and prediabetes symptoms.	Feasibility study	23	Chatbot: Tess	Dietary behavior
To et al, 2021 [71]	Australia	2021	To investigate the feasibility, usability, and effectiveness of a machine learning–based physical activity chatbot.	Quasi-experimental design	116	Mobile app/chatbot: Ida	Physical activity
Watanabe et al, 2023 [72]	Japan	2023	To preliminarily investigate (1) the effectiveness of the developed app in improving physical activity and reducing depression and anxiety, and (2) the app’s implementation outcomes, including acceptability, appropriateness, feasibility, satisfaction, and potential harm.	Nonrandomized trial	24 employees	Mobile app: ASHARE	Physical activity and mental health
Zahedani et al, 2023 [73]	United States	2023	To determine if a novel digital technology–based program, in which continuous glucose monitors and other health data points were used to provide individualized feedback and tailored recommendations based on a user’s personal data patterns, could improve lifestyle choices and metabolic health.	Retrospective cohort study	2217 people with diabetes	Mobile app: January AI app	Preventive care (blood glucose monitoring) and dietary behavior

a AI: artificial intelligence.

These studies evaluated the use and effectiveness of different types of AI systems (mobile apps, software, websites, platforms, devices, and chatbots) in various health promotion domains (dietary behavior, physical activity, smoking cessation, mental health, and preventive care). The most frequently used AI systems were mobile apps (14/22) and chatbots (9/22), while websites (5/22), software (2/22), and platforms (1/22) were less common. Notably, 6 of 22 studies used a combination of two types of AI systems, for instance, the combination of software and a website or a mobile app and a chatbot [53,54,56,64,65,68,71]. Regarding health promotion domains, 10 studies focused on dietary behavior, 6 related to smoking cessation, 4 focused on physical activity, 3 on mental health, and 3 on preventive care. Additionally, 4 studies incorporated multiple domains within a single AI-based intervention, integrating aspects like diet and physical activity or smoking cessation and mental health [52,64,65,72,73] (see Table 2, Table 3, and Figure 3).

**Table 3.** Distribution of different types of artificial intelligence systems.

Type	Frequency	Percentage
Other	2	7
Platform	1	4
Software	2	7
Chatbot	5	17
Website	5	17
Mobile app	14	48

### Primary Outcomes Measured

The primary outcome of this review was the impact of AI initiatives on the health promotion domain. Table 4 presents an overview of health promotion domains considered in each study. Disease reduction was directly targeted in studies about the self-management of hypertension [52], diabetes [56,73], obesity [63], and both obesity and prediabetes or diabetes [62,70]. All the studies reported positive impacts of the AI-based intervention on each domain of health promotion, although 10 of them reported uncertain results, and 3 of them also reported negative results.

**Table 4.** Overall impacts of artificial intelligence initiatives in health promotion and disease reduction.

Studies	Health promotion domain					Disease reduction (condition)	Overall impacts	Specific impacts		
	DB^a^	PhA^b^	SC^c^	MH^d^	PC^e^			Knowledge-cognition-behavior	Health status	Process (eg, usability, engagement)
Amiri et al, 2023 [51]	✓					N/A^f^	Creating meal plans tailored to the user’s individual needs and preferences Optimizing the nutrient value, providing better nutrition for the participants Addressing health-related nutrition intake (eg, controlling sodium intake)	N/A	N/A	‡^g^Optimized meal plan ‡User satisfaction
Branch et al, 2022 [52]	✓	✓		✓		DM^h^: hypertension	A significant drop in mean SBP^i^ following 3 months No change in SBP from 3 to 6 months for those who provided readings at both time points Lowering participants’ SBP by at least 1 classification category (eg, hypertension stage 2 to hypertension stage 1; hypertension stage 1 to elevated) by month 3	N/A	*^j^Lower SBP at 3 months †^k^Lower SBP at 6 months *Weight lost at 3 months	N/A
Brinsley et al, 2023 [53]				✓		N/A	Improvements in psychological distress, depression, anxiety, well-being, and return to work confidence Increased proportion of participants working at the end of the 6-week intervention	N/A	*Lower distress, anxiety, and depression scores *Higher well-being *Higher proportion of participants working	‡High completion and engagement rates
Brown et al, 2023 [54]			✓			N/A	Simply asking relevant questions about smoking was sufficient to confer benefits on the confidence attribute of the readiness ruler Extended conversation with improved generative reflections associated with a significant increase in average importance and readiness to quit.	*Increased importance of quitting smoking *Increased readiness to quit smoking †Number of quit attempts	N/A	N/A
Bucher et al, 2022 [55]					✓	N/A	Association with scheduling and attending mammograms for patients who were significantly overdue for recommended screening.	25% of people scheduled an appointment 88% of those who scheduled an appointment attended a mammogram	N/A	‡81.5% of people opened at least 1 message
Bul et al, 2023 [56]	✓					DM: diabetes	Well-received Primarily supported people with diabetes and their carers in identifying healthy recipes	N/A	Weight and waist circumference tended to decrease	‡Platform perceived as accessible and easy to use ‡Platform perceived as less useful for supporting meal planning and creating shopping lists
Carrasco-Hernandez et al, 2020 [57]			✓			N/A	Effectively supported smoking cessation by providing support and advice for facilitating abstinence, enhancing motivation, and clearly showing a benefit	*Smoking abstinence †Physical activity	†Lower BMI^l^ †Higher quality of life	N/A
Chen et al, 2021 [58]			✓			N/A	Promoting smoking cessation through user feedback	†The trend of increased cessation rate with increased response rate †More positive ratings of messages	N/A	‡User engagement was heterogeneous ‡73% of messages were rated as influential or very influential
Danieli et al, 2021 [59]				✓		N/A	Acceptable to mental health professionals (therapists) and users Therapists’ engagement in the participatory design model adopted in this study was favorable Increasing engagement of patients in the pursuit of their therapeutic goals	N/A	†Stress, distress, depression, and anxiety tended to decrease	N/A
Faro et al, 2023 [60]			✓			N/A	No significant difference in 6-month smoking cessation between groups Smoking cessation was significantly higher in viral tool kit versus no viral tool kit groups	†No difference in quit rate with artificial intelligence–generated messages *The quit rate increased in the artificial intelligence *Peer recruitment group	N/A	N/A
Forman et al, 2019 [61]	✓					N/A	Feasibility and acceptability of the method for preventing dietary lapses Effectiveness in reducing unplanned dietary lapses and facilitating weight loss	N/A	*Lower BMI and weight loss	‡Mean adherence to surveys was 85.1% ‡App rated as easy to use, useful, and enjoyable ‡Adherence dropped over the course of the study
Graham et al, 2022 [62]	✓					DM: obesity and type 2 diabetes	Interacting with a conversational artificial intelligence coach and weigh-ins were independently associated with a higher likelihood of achieving weight loss during the program Facilitating clinically meaningful reductions in body weight that can delay or prevent progression to type 2 diabetes	N/A	*Greater weight loss in the adherent group	*Higher engagement with artificial intelligence chatbot increased the likelihood of weight loss
Hassoon et al, 2021 [63]		✓				DM: obesity	Increasing steps in the MyCoach arm by an average of 3618.2 steps/day; Significantly greater net gain compared to the control arm and compared to SmartText	*Increased number of steps	N/A	N/A
Maher et al, 2020 [64], and Davis et al, 2020 [65]	✓	✓				N/A	High dietary adherence across all food groups Participants’ exceedance on their step goals 100% level of formality acceptable Comfortable chatting with Paola on Slack Negative comments about Paola provided by participants	*Increased physical activity *Adherence to the Mediterranean diet	*Weight loss and decrease in waist circumference	‡Frustration with the chatbot
Nakata et al, 2022 [66]	✓					N/A	Effective in promoting body weight reduction No significant differences in dietary intake and physical activity over 3 months Did not demonstrate the effectiveness of blood biochemistry measures A modest degree of weight loss was observed, which may enhance individual health	†Physical activity	*Weight loss †Biochemical markers	N/A
Okaniwa et al, 2022 [67]	✓					N/A	Human intervention with artificial intelligence and video messaging significantly reduced body fat percentage after 3 months The reduction was greater with more individualized intervention, with human video messages, and artificial intelligence text messages Challenging to sustain participants’ healthy behavior with artificial intelligence intervention alone.	*Smoking abstinence †After adjusting for confounders *More intensive use of the chatbot led to higher abstinence rates	N/A	‡61.2% of users accessed the chatbot
Olano-Espinosa et al, 2022 [68]			✓			N/A	Higher biochemically validated smoking abstinence rate at 6 months in the IG^m^ compared to the CG^n^ No substantial changes in adjusting for basal CO-oximetry and bupropion intake	N/A	*Reduced BFP^o^ *Artificial intelligence–based text messages reduce BMI †No significant effect on BFP	*Combined artificial intelligence and video messaging led to a lower dropout rate *Adding human intervention to artificial intelligence intervention achieved better persistence and health promotion effects
Perski et al, 2019 [69]			✓			N/A	Increased engagement and confidence Greater odds of quit success	*Smoking abstinence	N/A	*The addition of a chatbot led to a 101% increase in engagement
Stephens et al, 2019 [70]	✓					DM: obesity and prediabetes in youths	Experiencing positive progress toward participants’ goals Adolescents engaged with and viewed the chatbot as a helpful and usefulness app	N/A	N/A	‡Adolescents reported experiencing positive progress toward their goals 81% of the time ‡They reported usefulness ratings 96% of the time
To et al, 2021 [71]		✓				N/A	Usability of the chatbot and the Fitbit as at least “OK” Increased participants’ recorded steps and total physical activity, leading to them becoming more active Participants met more physical activity guidelines	*Average number of steps *Total physical activity minutes	N/A	‡87.6% of participants scored the usability of the chatbot as at least “OK” ‡35.4% would continue to use the chatbot in the future
Watanabe et al, 2023 [72]		✓		✓		N/A	The number of participants with severe psychological distress decreased significantly No significant improvements in physical activity levels or psychological distress among the participants	†Physical activity	*Reduced psychological distress	‡Acceptability, appropriateness, and satisfaction scores were lower than those in previous studies
Zahedani et al, 2023 [73]					✓	DM: type 2 diabetes	Improvements in metabolic health, healthy lifestyle practices, and weight management among participants Enhanced health outcomes that can prevent chronic conditions like type 2 diabetes	*Healthy eating habits; reduced daily caloric intake and carbohydrate-to-calorie ratio; increased intake of protein, fiber, and healthy fats	*Hyperglycemia, glucose variability, and hypoglycemia improved *Body weight decreased in all groups, especially those who were overweight or obese	*Healthy eating habits; reduced daily caloric intake and carbohydrate-to-calorie ratio; increased intake of protein, fiber, and healthy fats

a DB: dietary behavior.

b PhA: physical activity.

c SC: smoking cessation.

d MH: mental health.

e PC: preventive care.

f N/A: not applicable.

g ‡: descriptive findings.

h DM: disease management.

i SBP: systolic blood pressure.

j ∗: statistically significant positive impact.

k †: no significant impact; impact.

l BMI: body mass index.

m IG: intervention group.

n CG: control group.

o BFP: body fat percentage.

Specific impacts reported were grouped into 3 categories (knowledge-cognition-behavior, health status, and process), as depicted in Table 4. Smoking cessation had the most reported impact, with 4 studies showing statistically significant positive outcomes related to AI [57,60,68,69]. Physical activity and dietary behavior also showed positive impacts, with 3 [63,64,71] and 2 [64,73] studies with statistically significant positive outcomes, respectively. Regarding health status outcomes, 6 studies reported statistically significant impacts of AI interventions on weight loss [53,60,62,64,66,73], 2 on mental health dimensions [53,72], 1 on glycemic control [73], and 1 on systolic blood pressure [52]. However, some of these impacts were not sustained over time, as reported [61,67].

Process outcomes were generally assessed through descriptive methods. Most studies showed that AI-mediated interventions increased participant engagement, adherence, and satisfaction [51,53,58,61,62,68,69,71,72]. Ease of use, usefulness, and enjoyment were often reported with AI interventions [55,56,58,61,67,70,71]. However, some studies reported more negative experiences with the use of AI interventions such as chatbots [56,64,65]. Decrease in engagement over time, frustration regarding the interaction with the chatbot, and the need for human interaction were identified as potential drawbacks of AI-supported health promotion interventions [56,61,64,65,71,72].

### Secondary Outcomes Measured

Using thematic analysis, we categorized challenges, benefits, and suggestions into several subgroups. Challenges are presented in 5 subcategories: Ineffectiveness, Humanity and Specialty Needs, Lack of Engagement, Technical Limitations, and Unqualified Data. Benefits are divided into Practical Impacts, Positive Encouragement, Clinical Process Improvement, and Cost Effectiveness. In addition, suggestions are categorized into 5 subgroups: Future Investigation, Stakeholder Collaboration, End User Consideration, Technical Improvement, and Resource Allocation. The details of these categories are provided in Multimedia Appendix 3.

We used a SWOT matrix to present and analyze these results. For this, we focused separately on internal factors that strengthen or weaken AI implementation in health promotion and disease reduction, as well as external factors that create opportunities and threats for this context. Therefore, in this way, these 4 dimensions can be defined as follows:

1. Strengths (S): superior skills and resources that enhance the performance of AI systems in health promotion and disease reduction.
2. Weaknesses (W): deficiencies that can hinder the success of AI implementation in health promotion and disease reduction.
3. Opportunities (O): external trends that can improve the performance of these systems in promoting health or reducing disease if leveraged.
4. Threats (T): environmental factors that may decrease the performance of these systems in promoting health or reducing disease if not addressed.

The SWOT matrix is presented in Textbox 1. Internal challenges are defined as weaknesses and external ones are defined as threats. Similarly, the internal benefits are categorized as strengths and external ones as opportunities.

Textbox 1. **SWOT matrix of the extracted challenges, benefits, and suggestions of artificial intelligence implementation.**

**Strengths (S)**

***Practical impacts***

- Alleviate the drawbacks of behavioral interventions [57]
- Personalized coach and feedback [62]
- Practical and supportive interventions for users [53]

***Positive encouragement***

- Considerable interest in virtual health coaches as a care supplement [53]
- High contact number and favored long interaction [68]
- Providing feedback and progress [62]

***Clinical process improvement***

- Limited impacts on clinician workflow [55]
- Removing additional steps for providers by automating processes [55,62]
- Considering the providers’ capacity in scheduling communication time and frequency [55]

***Cost-effectiveness***

- Cost-sensitive algorithms [61]

**Weaknesses (W)**

***Ineffectiveness impact***

- Poor evaluation of the artificial intelligence implementation outcomes [72]
- Lower accuracy of the self-reported measurement [54]
- No significant difference in results [57]
- The lack of a full explanation of the results [66]

***Humanity and specialty needs***

- Requiring human communication process [67]
- Frequent checking of input by the provider [66]

***Lack of engagement***

- The lack of participant engagement [54,56]
- Feeling there is no added value [56]
- Less relevance of the platform over time [56]
- No exploration of the effective factors on unsubscribed participants [55]

***Technical limitations***

- Insufficient function in collecting users’ votes [57]
- Not predicting the best time for message-sending [57]
- Not accessing older data or diagnosis history [57,62]
- The existence of lapses in the outcome [61]
- Limitation of dissemination and implementation due to the edition language [66]

***Unqualified data***

- Modification of rating value by users [57]
- Inconsistent values and inaccurate data [57]
- Removing some data and values [57]
- No access to demographic information [62]

**Opportunities (O)**

***Practical impacts***

- Accessibility, simplicity, ubiquity, and immediacy [68]
- Facilitating patient access to high-quality treatments [68]
- Death and illness prevention [54,68]
- Helping people quit smoking sooner [54]
- Applicability of the platform in diabetes management [56]
- Improving psychosocial outcomes [53]

***Positive encouragement***

- Encouraging self-tracking and engagement [62]
- Motivating patients to have a healthy diet [56]
- Encouragement in scheduling and attending mammograms [55]
- Increasing self-monitoring and self-reported exercise [62]
- Improving the rate of return-to-work outcomes [53]

***Clinical process improvement***

- Workload reduction of health professionals [68]
- Maximizing the throughput of mammography schedules [55]
- Not adding stress for providers regarding monitoring and adjusting mammography schedules [55]
- Easy dissemination in health care settings due to it being free to download [66]

***Cost-effectiveness***

- Mitigating the excess demand for screening centers [55]
- Cost savings for health providers [68]
- Providing low-cost interventions [53]

**Threats (T)**

***Ineffectiveness impact***

- Short-term effects of text message reminders [67]
- Short-term effects of the provided information [67]

***Humanity and specialty needs***

- Less effective in conveying health information [67]
- Requiring health professionals for medication prescription [68]
- Needing dietitians to moderate the accuracy of the provided information [56]
- Increasing the provider workload in patient-directed behavioral interventions [55]
- Diabetes experts’ debate on the usefulness of platforms over time [56]

***Lack of engagement***

- Rational world bias in ongoing healthy behavior and long-term health improvement [67]

### Weaknesses and Threats

The most challenging factors in AI implementation in health promotion were identified as the short-term impacts of AI interventions in promoting health [54,57,66,67,72], requiring human intervention and specialization of providers [55,56,66-68], lack of participant engagement in related studies [54-56,67], technical limitations of AI-based systems (eg, lack of access to past clinical data) [57,61,62,66], and concerns about data quality [57,62]. In categorizing the challenges, deficiencies in the performance of AI initiatives and the lack of optimal evaluation of them are considered weaknesses. On the other hand, external negative impacts, such as the short-term implications of AI initiatives, the requirement for expert interpretation of the outputs, and the lack of consensus on improving health and healthy lifestyles, are seen as threats.

### Strengths and Opportunities

Along with the challenges, this study also highlighted the benefits of using AI to promote health and reduce disease. These include facilitating patient access [54,68] by providing straightforward and applicable systems [54], the improvement of personalized coaching with detailed feedback, preventive care [53,54,57,62,68], encouraging people to incorporate healthy behaviors [53,55,56,62,68], clinical process and workflow improvement [55,62,66,68], and providing cost-effectiveness interventions [53,55,61,68]. When discussing the benefits, internal ones such as providing feedback [53,62,68], reducing the drawbacks of interventions [53,57,62], and using cost-sensitive algorithms [61] are considered strengths. External positive impacts, such as providing accessible interventions [53,54,56,68], motivating people to self-track and live healthy [53,55,56,62], not creating stress for providers [55], reducing the providers’ workload [55,66,68], and mitigating excess demand [53,55,68], are seen as opportunities.

### OS, OW, TS, and TW Strategies

The identified recommendations include more investigation into the different aspects of AI application in health promotion, such as economic aspects [51,53,55,56,69], the collaboration between diverse stakeholders [56,57,59], considering end users’ needs and preferences in planning and designing AI-based interventions [56,57,59,64,67,72,73], enhancing technical performance and features of the AI initiatives [51,54,61,62,64,68,69], and allocating sufficient financial and human resources [64,69]. By using SWOT analysis, we display suggestions across 4 categories: OS (Opportunities, Strengths), OW (Opportunities, Weaknesses), TS (Threats, Strengths), and TW (Threats, Weaknesses) (Table 5). To explain further, using strengths such as the cost-effectiveness of AI algorithms can enhance the opportunities for exploring different aspects of AI. In addition, the benefits of saving money and reducing costs can lead to more technical advancements in AI systems, achievable through collaboration with key stakeholders. This approach can not only improve weaknesses but also open up new opportunities.

**Table 5.** Proposed strategies to advance AI^a^ implementation in health promotion and disease prevention.

Type	Strategies
OS^b^	**Further investigation** Identifying effects by conducting randomized controlled trials or quasi-experimental implementation [55,61] Investigating the economics of a behavioral intervention [55] **End user consideration** Improving acceptability, appropriateness, and satisfaction [56,72] **Technical improvements** Using personalized technical assistance to facilitate accessibility by users [68] Assessing the AI initiative’s performance via various mechanisms [69] Gathering more comprehensive feedback from users [51,62] Evaluating the app’s usefulness, usability, and overall user satisfaction [51] Objective tracking to be aware of one’s lifestyle behaviors [62]
OW^c^	**Further investigation** Exploring the effect of conversational agents on behavioral and experiential engagement indicators [69] Capturing details of process outcomes to improve program elements (eg, language style) [53] Designing longitudinal studies to capture long-term effects and health outcomes in lifestyle behaviors [51,56] Examining the role of motivation and self-efficacy in interventions [56] **End user consideration** Designing and improving AI according to the users’ needs, preferences, experiences, and expectations [56,59,64] Involving end users and therapists in the design process [59] Considering the needs of different age groups [73] Reviewing the recipe content by diabetes experts to provide a reliable and healthy personalized diet [56] Needing the careful analysis of user engagement across time [57] **Technical improvements** Updating development to adapt to the user’s needs and circumstances [51] Facilitating continued compliance over time and using data [61] Assessing the app’s performance in real-world scenarios [51] Initiating regular conversations between users and the AI system [64] Conducting usability tests for AI-based interventions [59] **Resource allocation** Allocating enough resources to support intensive and ongoing trials [64,69] Requiring substantial expertise and time [69]
TS^d^	**Stakeholder collaboration** Co-creating solutions with people and health care professionals in further AI development [56] **End user consideration** Considering the people’s preference for interventions [67] Requiring the process of person-to-person communication for health interventions [67] Knowing the intervention types that encourage people in health activities [67] Optimizing user engagement by customization to varied ethnicities and socioeconomic levels [73] **Technical improvements** Using more algorithms and real-time data sources to generate effective responses and reflections [54,71] Incorporating the AI therapeutic solution into the health care providers’ usual care [57]
TW^e^	**Further investigation** Testing and validating AI algorithms in a clinical setting on a larger sample [59,63,71] Investigating the clinical and statistical impact of the system prediction [61] Conducting a confirmatory trial to help disseminate and implement the AI system [66] **Stakeholder collaboration** Requiring collaboration between different stakeholders to design user-friendly and clinically relevant AI [59] **End user consideration** Designing a more complex, longer conversation, considering more aspects of a clinician-delivered conversation [54] Considering visual impairments, ethnicity, and socioeconomic status in designing AI systems [56] Considering different preferences in terms of delivery mode among participants [56] **Technical improvements** Paying attention to produce a more natural and less robotic conversational style [64]

a AI: artificial intelligence.

b OS: Opportunities, Strengths.

c OW: Opportunities, Weaknesses.

d TS: Threats, Strengths.

e TW: Threats, Weaknesses.

## Discussion

### Principal Findings

We conducted this rapid knowledge synthesis to address the growing demand for evidence on the practical implementation of AI-based initiatives in promoting health and reducing diseases. Our review highlights several benefits of integrating AI into health promotion and chronic disease self-management interventions, both of which contribute directly to disease reduction. AI interventions showed significant improvements, particularly in promoting smoking cessation, physical activity, and dietary patterns.

Our research findings align with the extensive evidence demonstrating the efficacy of AI initiatives in promoting health and reducing chronic diseases [74]. Our analysis contributes to the growing body of evidence on the efficacy of AI in enhancing self-management for individuals living with hypertension, prediabetes, diabetes, and obesity [54,56-58,61,66,69,73]. A systematic review has also demonstrated the efficacy of AI chatbots in areas such as promoting healthy behavior and lifestyles, smoking cessation, and medication adherence [20]. Several AI-driven interventions have demonstrated significant success in real-world applications. For instance, AI-powered chatbots have effectively facilitated smoking cessation by providing personalized motivation and behavioral feedback [69,75]. Additionally, mobile-based AI interventions have shown positive impacts on promoting physical activity by offering adaptive goal-setting and real-time coaching, leading to improved adherence to exercise routines [63,76]. Although both types of interventions showed promise, chatbot-based solutions appeared particularly effective for initiating behavior change, whereas mobile apps were better suited for sustaining long-term adherence. However, the heterogeneity in study designs and outcome measures prevents definitive comparisons between intervention types. These patterns indicate the potential for targeted use of specific AI modalities, but more head-to-head studies are needed to establish comparative effectiveness.

Our review also shows that AI has the potential to influence the health status of the population by aiding in weight loss and mental health; however, its unsustainability over time is underscored. In line with our findings, it is reported that AI has the potential to aid in weight loss [77] and enhance mental health [78], but its use is still undeveloped, and the ethical challenges must be addressed [77,78]. AI-based interventions also yielded significant improvements in the process outcomes, leading to increased participant engagement, adherence, and satisfaction. It is, however, important to note that there were documented instances of adverse experiences that manifested in reduced engagement over time and frustration during interaction [56,64,65]. Certain AI interventions struggled to sustain long-term behavior change, possibly due to the novelty effect or limited personalization. Some studies also reported challenges in adapting AI tools to users’ evolving needs and a lack of follow-up assessments to confirm lasting effects [20,32,79]. To address these challenges, incorporating strategies from behavioral science and user experience design, such as gamification, adaptive feedback loops, and interactive AI coaching, could improve long-term user engagement and adherence to AI-driven health interventions [80-82]. Limited direct comparisons with traditional non-AI interventions suggest that AI-powered tools may enhance personalization and accessibility in ways that conventional methods cannot, particularly in remote monitoring and behavioral coaching [83,84]. However, further research is needed to compare the long-term efficacy and user satisfaction between AI-driven and traditional health promotion strategies to determine their relative advantages and potential complementarities.

Regarding the overall experiences of using AI in health promotion, previous reviews have highlighted the impact of AI interventions on enhancing participant involvement and their high usability and acceptability in health-related activities [20,85,86]. Beyond the findings of this review, AI has significant potential to enhance health care delivery in low-resource and underserved settings [87]. AI-driven tools can bridge gaps in access by enabling remote monitoring, telehealth, and automated decision support, particularly in areas with workforce shortages. Mobile AI apps and chatbots could also improve access to evidence-based health information, helping individuals overcome barriers related to geography, cost, and availability of care [88,89]. However, the scalability of AI-based interventions in rural and low-income settings depends on several key factors, including infrastructure availability, digital literacy, and integration with existing health care workflows [90,91]. Without proactive efforts to bridge these gaps, AI could inadvertently reinforce existing health inequities. Therefore, equity-oriented implementation strategies—including localized adaptation, capacity building, and inclusive design—are essential to ensure that AI benefits are equitably distributed. Indeed, for sustainable implementation, AI solutions must be tailored to the specific needs of underserved populations, ensuring that technological advancements do not inadvertently widen health disparities [91,92]. Future initiatives should prioritize context-specific adaptations and capacity-building efforts to maximize the reach and effectiveness of AI-driven health interventions.

However, it has been discussed that AI can raise concerns about trust, responsibility, and emotional engagement [93]. These concerns are particularly relevant in mental health applications, where ensuring ethical safeguards and human oversight is essential to building trust in AI-driven interventions [94,95]. Although our review identified key ethical and technical challenges associated with AI in health promotion, additional challenges must also be considered. Data privacy, participant consent, and algorithmic bias are critical considerations in AI-driven health care. Although AI can enhance engagement and accessibility [53,62,68], concerns remain regarding data security, informed consent, and the potential misuse of sensitive health information [96,97]. Biases in training datasets may also lead to inequitable health outcomes, reinforcing disparities in underserved populations [41,98]. These biases can disproportionately affect certain demographic groups based on factors such as gender, age, socioeconomic status, and cultural background [99,100]. Addressing these challenges requires transparent data governance, adherence to regulatory frameworks (eg, General Data Protection Regulation, Health Insurance Portability and Accountability Act), and robust bias mitigation strategies [101-104]. Additionally, implementing explainable AI and strengthening cybersecurity protocols can help ensure fairness, user trust, and ethical AI integration in health care [105,106]. Furthermore, the development of inclusive smart health models, the enhancement of data diversity, the application of debiasing techniques, and the adoption of human-centered approaches that prioritize the needs of marginalized communities are recommended to mitigate these issues [99,107]. This demonstrates that the integration of AI in health care remains a nascent emerging field requiring more transparent and trustworthy implementation [108].

We also identified the related challenges, benefits, and suggestions from the included studies and analyzed them using a SWOT analysis. According to the SWOT analysis, the predominant strengths include providing supportive coaching and feedback for users, achieving a high rate of participation, and improving clinical processes. These favorable attributes of AI are consistent with other research on the benefits of allowing users to receive personalized care and guidance in managing and controlling their health status [109], and engaging more in persuading their care plan [110], all without adding extra burdens on clinical procedures [111]. The main weaknesses identified were mostly related to technical limitations, unqualified data, and human aspects. In comparison to related studies, our findings align with previous reviews, emphasizing the constraints of insufficient data, poor validation, ethical concerns, legal barriers [23,112,113], and technical obstacles [114].

The identified opportunities encompassed aspects such as patient motivation, reduction of health professionals’ workload, and interventions aimed at cost-saving. The findings are in line with previous research, underscoring the important role of AI in decreasing the workload of health professionals, addressing the workforce shortage [115], improving patient engagement and comprehension [116], and reducing health care costs [117]. The main threats were the imperative for human involvement in results interpretation and communication, along with the short-term impacts of AI-based interventions. Corresponding with our results, Udegbe’s review [118] revealed the intricacies of human-AI interaction as an important obstacle in AI implementation in health care. Furthermore, the limited generalizability, the need for robust clinical evaluation [119,120], and problems with sustainable usability and efficacy [32,33] were cited as critical challenges in implementing AI for promoting health and reducing diseases.

Our analysis adds to the existing evidence by providing practical recommendations for using AI, specifically in health promotion and disease reduction strategies. The key identified strategies include conducting further investigations, taking into account the needs of end users, improving the technical aspect of the technology, and allocating resources. It is recommended that mitigating the weaknesses of AI in health promotion, such as technical limitations and data quality concerns, includes structured interdisciplinary collaboration [121]. Engaging health care professionals ensures that AI applications align with clinical workflows and patient needs. Data scientists and AI engineers contribute by refining algorithmic accuracy and robustness through improved data preprocessing and validation techniques. Ethicists and policymakers play a crucial role in establishing governance frameworks that enhance data transparency and security while addressing potential biases. Moreover, integrating public health experts and community representatives into AI development can improve dataset diversity, ensuring AI systems are trained on representative populations. By fostering structured interdisciplinary collaboration, AI tools can become more reliable, equitable, and aligned with real-world health care challenges [122,123]. In alignment with these recommendations, several guidelines and road maps have been published. Although these focus on AI implementation in health care systems, they can serve as a guiding light for leveraging AI to promote health and mitigate diseases. These tool kits offer best practices for the implementation of AI in health care, address key challenges in AI adoption, offer recommendations for optimizing AI deployment in health systems [124], and recommend practical guidance for AI governance to help maximize the benefits of AI while minimizing its foreseeable risks [125].

### Potential Impact and Future Directions

To harness the potential of AI in health promotion and disease reduction, recent advancements in AI technologies should be acknowledged. Innovative AI tools, such as AI-based virtual health assistants (eg, Lucy [126], Paola [65]), interactive chatbots (Woebot [127], Replika [128]), digital health coaching systems (Lark [129], Ginger [130]), and diagnostic apps (Ada Health [131], Babylon Health [132]), are emerging as promising solutions for enhancing personalized health advice, supporting behavior change, and improving chronic disease management. Highlighting such advancements can inspire future research to explore their broader applicability in diverse health care settings. Future studies should focus on assessing the effectiveness, scalability, and ethical implications of these technologies in real-world contexts.

First, we believe that to facilitate using AI in the field of health promotion and disease reduction, it is essential to conduct real-world studies to assess the effectiveness and long-term sustainability of AI interventions across diverse populations, given the observed benefits in areas like smoking cessation, physical activity, and dietary improvement. Second, addressing user engagement and trust issues is critical; although AI interventions have demonstrated high usability, concerns around trust and emotional connection highlight the need for transparent, user-centered design to enhance acceptance and lead to consistent engagement. Finally, economic evaluations are crucial to determine the cost-effectiveness and scalability of AI applications in health care systems, as these interventions have shown promise in reducing health care costs and the workload of health professionals. Future studies should incorporate structured economic evaluations, such as cost-benefit and cost-effectiveness analyses, to assess the financial viability of AI-driven health interventions and guide resource allocation and inform policy decisions on sustainable AI adoption.

In addition to these recommendations, the seamless integration of AI technologies into existing health care workflows is essential to maximize their benefits. Policymakers and health care stakeholders should prioritize regulatory frameworks that support the ethical and equitable adoption of AI while ensuring alignment with clinical guidelines and public health priorities. It is worth mentioning that effective AI integration requires collaboration among policymakers, health care providers, technology developers, and public health officials to address ethical, technical, and infrastructural challenges. AI initiatives should be designed with interoperability in mind to facilitate adoption in diverse health care settings. These findings align with global digital health strategies, such as those outlined by the World Health Organization, which emphasize the role of AI in strengthening health care systems, improving accessibility, and supporting data-driven decision-making. However, future research should further explore policy-driven approaches to ensure AI adoption is both sustainable and ethically sound across different health care contexts.

### Limitations of the Study Design

The main limitation of this review is its design. Contrary to systematic reviews, but consistent with protocols for expedited reviews [37], we narrowed the scope of the search to align with the study’s objectives by using limited databases and imposing a publication date restriction. These methodological choices allowed for appropriate and structured study selection, data extraction, and critical appraisal in a timely manner. However, it is important to note that this rapid review has limitations.

To ensure that stakeholders and decision-makers receive the most current evidence, the search was restricted to studies published in the last 5 years and limited to Organisation for Economic Co-operation and Development countries. Although this approach enhances relevance to contemporary AI applications, it may affect the generalizability of findings, particularly to low- and middle-income countries, where health care infrastructure, digital literacy, and AI accessibility differ significantly. Additionally, several included studies had small sample sizes, which may limit the robustness and generalizability of the findings. The reliance on small cohorts can introduce selection bias and reduce the statistical power needed to validate AI-driven interventions in diverse populations.

Moreover, the review may be subject to publication bias, as studies reporting positive outcomes are more likely to be published than those with null or negative findings. Although we acknowledged this risk, we did not incorporate gray literature or preprints, which could have improved the comprehensiveness of our evidence base. Therefore, future research should expand its scope by incorporating a broader range of countries, more diverse databases, and larger, more representative samples. Future reviews should also integrate mitigation strategies, such as the inclusion of gray literature, to enhance methodological rigor and reduce potential publication bias.

Furthermore, no formal quality appraisal or risk of bias assessment was conducted for the included studies. Although this is consistent with common practices in rapid reviews, it limits the ability to assess the strength and certainty of the synthesized evidence. Future studies should incorporate standardized quality assessment tools to improve the credibility of findings.

Lastly, while our review focused on the application of AI in health promotion and disease reduction, we acknowledge that a detailed technical analysis of AI models, including their accuracy, efficiency, and scalability, was beyond the scope of this study. Future research should explore these aspects, particularly by integrating causal inference methods such as propensity score matching, instrumental variable analysis, and difference-in-differences to establish the direct impact of AI interventions on health outcomes. Such approaches would provide stronger evidence on whether AI-based health interventions yield superior results compared to traditional methods. To bridge gaps in long-term outcomes and strengthen the evidence base for AI-driven health promotion interventions, future research should use robust study designs such as longitudinal studies and randomized controlled trials. These methodologies would provide deeper insights into the sustained impact of AI interventions over time and improve causal inferences regarding their effectiveness.

### Conclusion

The integration of AI and related technologies holds considerable potential to enhance health promotion efforts and prevent chronic conditions through personalized, scalable, and interactive interventions. This review synthesized existing evidence and practical applications demonstrating the effectiveness of AI in supporting physical activity, dietary improvements, smoking cessation, and mental well-being across diverse populations. Importantly, the findings of this review highlight several critical priorities for future policy and implementation. First, personalization and real-time engagement are central to the success of AI-based interventions, though maintaining long-term user interaction and trust remains a key challenge. Second, while AI tools offer significant scalability and remote accessibility, their effectiveness in underserved communities is contingent upon addressing infrastructural and digital literacy barriers. Third, ethical concerns—including algorithmic bias, data privacy, and informed consent—must be proactively addressed through inclusive design, regulatory oversight, and transparent governance mechanisms. Additionally, the lack of long-term impact assessments and comparative effectiveness studies underscores the need for robust real-world research and economic evaluations to guide sustainable adoption. The SWOT analysis presented in this review offers a strategic framework to inform implementation pathways. By aligning AI deployment with health care system priorities and involving multidisciplinary stakeholders, including health care providers, public health professionals, developers, and policymakers, AI can be more effectively integrated into clinical and community-based health promotion strategies. This synthesis thus provides a foundation for designing equitable, efficient, and ethically sound AI-driven health interventions and supports decision-makers in translating AI innovations into improved population health outcomes.

## Supplementary material

10.2196/70381 Multimedia Appendix 1 Member countries.

10.2196/70381 Multimedia Appendix 2 Search strategy.

10.2196/70381 Multimedia Appendix 3 Data synthesis.

## Abbreviations

AI: artificial intelligence
MeSH: Medical Subject Headings
PRISMA: Preferred Reporting Items for Systematic Reviews and Meta-Analyses
SWOT: Strength-Weakness-Opportunity-Threat
