# Supplementary material for: Artificial Intelligence in Health Promotion and Disease Reduction: Rapid Review
Source: J Med Internet Res. 2025 Aug 1;27:e70381. doi: 10.2196/70381 (PMC12337235; doi:10.2196/70381)
Supplement: Multimedia Appendix 1 [file jmir-v27-e70381-s001.docx]

**Appendix 1: OCED Countries**

As of May 2021, there are 38 members of the OECD:

| Australia | Costa Rica | Germany | Italy | Netherlands | Slovenia | United Kingdom |
| --- | --- | --- | --- | --- | --- | --- |
| Austria | Czech Republic | Greece | Japan | New Zealand | South Korea | United States |
| Belgium | Denmark | Hungary | Latvia | Norway | Spain |  |
| Canada | Estonia | Iceland | Lithuania | Poland | Sweden |  |
| Chile | Finland | Ireland | Luxembourg | Portugal | Switzerland |  |
| Colombia | France | Israel | Mexico | Slovakia | Turkey |  |
