# Supplementary material for: Artificial Intelligence in Health Promotion and Disease Reduction: Rapid Review
Source: J Med Internet Res. 2025 Aug 1;27:e70381. doi: 10.2196/70381 (PMC12337235; doi:10.2196/70381)
Supplement: Multimedia Appendix 2 [file jmir-v27-e70381-s002.docx]

**Appendix 2: Search Strategy**

Medline (Ovid)

Date of the search: 15-03-2024

Database limit: results have been limited to the last 5 years only; editorials and reviews were excluded; animal studies were also excluded.

| # | Search strategy | Results |
| --- | --- | --- |
| 1 | Artificial Intelligence/ OR exp "Machine Learning"/ OR ((artificial OR computational) adj2 intelligence).ti,ab,kf OR ((Machine? OR Supervised OR Unsupervised OR Reinforcement OR Deep) adj2 Learning).ti,ab,kf OR "Neural Networks, Computer"/ OR "Neural Networks".ti,ab,kf | 266722 |
| 2 | Exercise/ OR (Physical adj2 (Activit* OR Exercise?)).ti,ab,kf OR "Diet, Healthy"/ OR Diet/ OR Feeding Behavior/ OR Sleep/ OR (Healthy adj2 (Diet? OR nutrition)).ti,ab,kf OR exp Sleep Hygiene/ OR (Sleep adj2 (Habit? OR qualit* OR Restorati* OR Hygiene)).ti,ab,kf OR (diet* adj1 (intake? OR quality OR pattern? OR habit? OR management OR change? OR lifestyle? OR behavior)).ti,ab,kf OR (Healthy adj2 (nutrition OR eat OR eating)).ti,ab,kf OR Health Behavior/ OR "behavior* change".ti,ab,kf OR (Feeding adj2 (Behavior? OR Pattern? OR Habit?)).ti,ab,kf OR "Stress, Psychological"/ OR (Stress adj2 Manag*).ti,ab,kf OR Smoking Cessation/ OR Smoking Reduction/ OR "Tobacco Use Cessation"/ OR ((Smoking OR Tobacco) adj2 ("Giving Up" OR Quitting OR Cessation OR Reduction)).ti,ab,kf OR Smokeless.ti,ab,kf OR (Alcohol adj2 (Drinking OR Consumption OR Intake) adj2 ("Giving Up" OR Quitting OR Cessation OR Reduction)).ti,ab,kf OR Life Style/ OR Healthy Lifestyle/ OR (("Life Style?" OR Lifestyle?) adj2 healthy).ti,ab,kf OR Health Promotion/ OR (Health* adj2 (promot* OR Campaign?)).ti,ab,kf OR Primary Prevention/ OR Secondary Prevention/ OR Tertiary Prevention/ OR Health Communication/ OR Health Education/ OR exp Consumer Health Information/ OR ((Health OR patient?) adj2 (communication OR education OR literacy)).ti,ab,kf | 1196389 |
| 3 | 1 AND 2 | 4814 |
| 4 | limit 3 to yr=2019-2024 | 3633 |
| 5 | Systematic Review/ OR Review/ OR Meta-Analysis/ OR meta-analysis as topic/ OR "Review Literature as Topic"/ OR "systematic review (topic)"/ OR review?.ti,kf OR Editorial/ OR Comment/ OR Letter/ OR editorial.ti | 5828028 |
| 6 | 4 NOT 5 | 3046 |
| 7 | 6 NOT (Animals/ NOT humans/) | 2901 |

CINAHL

Date of the search: 15-03-2024

Database limit: results have been limited to the last 5 years only; editorials and reviews were excluded; animal studies were also excluded.

| # | Search strategy | Results |
| --- | --- | --- |
| 1 | MH "Artificial Intelligence" OR MH "Machine Learning+" OR MH "Natural Language Processing" OR MH "Neural Networks (Computer)" OR TI ((artificial OR computational) N2 intelligence) OR AB ((artificial OR computational) N2 intelligence) OR TI ((Machine# OR Supervised OR Unsupervised OR Reinforcement OR Deep) N2 Learning) OR AB ((Machine# OR Supervised OR Unsupervised OR Reinforcement OR Deep) N2 Learning) OR TI "Neural Networks" OR AB "Neural Networks" | 40,046 |
| 2 | MH Exercise OR TI (Physical N2 (Activit* OR Exercise#)) OR AB (Physical N2 (Activit* OR Exercise#)) OR MH "Dietary Patterns" OR MH Diet OR MH "Eating Behavior" OR MH "Sleep" OR MH "Sleep Hygiene+" OR TI (Healthy N2 (Diet# OR nutrition)) OR AB (Healthy N2 (Diet# OR nutrition)) OR TI (Sleep N2 (Habit# OR qualit* OR Restorati* OR Hygiene)) OR AB (Sleep N2 (Habit# OR qualit* OR Restorati* OR Hygiene)) OR TI (diet* N1 (intake# OR quality OR pattern# OR habit# OR management OR change# OR lifestyle# OR behavior)) OR AB (diet* N1 (intake# OR quality OR pattern# OR habit# OR management OR change# OR lifestyle# OR behavior)) OR TI (Healthy N2 (nutrition OR eat OR eating)) OR AB (Healthy N2 (nutrition OR eat OR eating))  MH "Health Behavior" OR TI "behavior* change" OR AB "behavior* change" OR TI (Feeding N2 (Behavior# OR Pattern# OR Habit#)) OR AB (Feeding N2 (Behavior# OR Pattern# OR Habit#)) OR MH "Stress, Psychological" OR TI (Stress N2 Manag*) OR AB (Stress N2 Manag*) OR MH "Smoking Cessation" OR TI ((Smoking OR Tobacco) N2 ("Giving Up" OR Quitting OR Cessation OR Reduction)) OR AB ((Smoking OR Tobacco) N2 ("Giving Up" OR Quitting OR Cessation OR Reduction)) OR TI Smokeless OR AB Smokeless OR TI (Alcohol N2 (Drinking OR Consumption OR Intake) N2 ("Giving Up" OR Quitting OR Cessation OR Reduction)) OR AB (Alcohol N2 (Drinking OR Consumption OR Intake) N2 ("Giving Up" OR Quitting OR Cessation OR Reduction)) OR MH "Life Style" OR MH "Life Style Changes" OR MH "Health Promotion" OR MH "Health Education" OR TI (("Life Style#" OR Lifestyle#) N2 healthy) OR AB (("Life Style#" OR Lifestyle#) N2 healthy) OR TI (Health* N2 (promot* OR Campaign#)) OR AB (Health* N2 (promot* OR Campaign#)) OR MH "Consumer Health Information+" OR TI ((Health OR patient#) N2 (communication OR education OR literacy)) OR AB ((Health OR patient#) N2 (communication OR education OR literacy)) | 562,365 |
| 3 | S1 AND S2 | 1,338 |
| 4 | S3 AND PY 2019-2024 | 1,064 |
| 5 | MH ("Literature Review+" OR "Meta Analysis") OR TI review# OR TI editorial | 426,064 |
| 6 | S4 NOT S5 | 981 |
| 7 | S6 NOT (Animals/ NOT humans/) | 975 |
