# Supplementary material for: Artificial Intelligence in Health Promotion and Disease Reduction: Rapid Review
Source: J Med Internet Res. 2025 Aug 1;27:e70381. doi: 10.2196/70381 (PMC12337235; doi:10.2196/70381)
Supplement: Multimedia Appendix 3 [file jmir-v27-e70381-s003.docx]

**Multimedia Appendix 3. Data Synthesis**

Table 6) Summary of the Challenges, Benefits, and Suggestions for AI Initiatives in Health Promotion and Disease Reduction

| Main Themes | Sub-Themes | Items |
| --- | --- | --- |
| **Challenges:**  **Weakness (W) Threats (T)** | **Ineffectiveness Impact** | - The short-term effects of text messages by AI on promoting weight loss **(T)** (1) - Low effectiveness of AI because of the poor evaluation of the implementation outcomes by app users **(W)** (2) - The short-term effects of information provided through AI alone **(T)** (1) - The lower accuracy of the self-reported measures to evaluate chatbot than clinician-administered survey **(W)** (3) - No significant difference between successful participants in smoking cessation and not successful ones **(W)** (4). - The lack of a full explanation of body weight change neither by energy intake measured by BDHQ nor physical activity measured by the accelerometer **(W)** (5) |
|  | **Humanity and Specialty Needs** | - Less effectiveness in conveying health information by AI, compared to humans **(T)** (1) - Requiring a health professional for medication prescription, instead of AI direct intervention **(T)**(6) - The importance of the process of human communication rather than the information content **(W)** (1) - Needing dietitians to moderate the accuracy of nutritional information provided by AI **(T)**(7) - Increasing the provider workload by the patient-directed behavioral interventions **(T)** (8) - Diabetes experts debate the usefulness of web-based platforms in actual behavior change over time **(T)** (7) - The frequent check of the data input to enhancement of the intervention-group adherence by provider **(W)** (5) |
|  | **Lack of Engagement** | - The existence of a rational world bias in ongoing healthy behavior and long-term health improvement **(T)** (1) - The lack of participants' engagement in finishing the study **(W)** (3) - Feeling no added value and non-participation in using the platforms or filling the survey by participants **(W)** (7) - The feel of the less relevance of the platform over time for those who managed their diabetes relatively well **(W)** (7) - The challenges of high-level engagement over a long period of time with a web-based platform and study procedures **(W)** (7) - Not exploration of the demographics, engagement, and behavioral outcomes of unsubscribed participants to reduce the unsubscribe rate **(W)** (8) |
|  | **Technical Limitations** | - Insufficient function of the AI-based system in collecting users vote **(W)** (4) - The inability to predict the best time to send messages based on HRS by AI **(W)** (4) - The inability of the AI-based system to retrieve information from older data and to access medical information or diagnosis history **(W)** (4, 9) - The limitation of the dissemination and implementation of CALO Mama Plus in other countries because of its Japanese edition **(W)** (5) - The probable existence of any lapse (specific or general) in the future iterations of the eating plan by OnTrack **(W)** (10) |
|  | **Unqualified Data** | - Modification of rating value by participants, believing these rates are under the view of healthcare professionals **(W)** (4) - Inconsistent values and inaccurate entire data by participants in the entries of the registered time **(W)** (4) - Reducing the data quality by removing some data and values **(W)** (4) - Having limited access to demographic information **(W)** (9) |
| **Benefits:**  **Strength (S)**  **Opportunity (O)** | **Practical Impacts** | - Accessibility, simplicity, ubiquity, and immediacy of chatbots as an intervention in smoking abstinence **(O)** (6) - Facilitating patient access to high-quality treatments and preventing death due to tobacco smoking **(O)**(3, 6) - The strong potential of chatbots to help sooner quit smoking, resulting in illness prevention **(O)**(3) - The more applicability of the platform for people, who have difficulties with diabetes management or are unaware of their eating habits impact **(O)** (7) - The alleviate of drawbacks of intensive complementary behavioral interventions in smoking cessation by digital therapeutic solution **(S)** (4) - The Improvement of the personalized coaching and feedback by AI Coach’s mechanism with learning the patterns and preferences **(S)** (9) - Providing practical and supportive interventions for active workers’ compensation claims by AI **(S)** (11) - Improving psychosocial outcomes among individuals on an active workers’ compensation claim by AI **(O)** (11) |
|  | **Positive Encouragement** | - The considerable interest in virtual health coach intervention, as a supplement to usual care **(S)** (11) - The high contact number and favored long interaction with a chatbot as an intervention in smoking abstinence **(S)** (6) - Encouraging self-tracking and engagement by “nudges” or reminders, by AI-Coach **(O)** (9) - The benefits of the platform for people with long-term health conditions to have healthy nutrition and weight loss in preventing diabetes **(O)** (7) - Women's and patients’ encouragement in scheduling and attending mammograms by using digital health intervention (CALO Mama Plus) **(O)** (8) - Increasing the self-monitoring and self-reported exercise by AI-Coach reminders **(O)** (9) - Improving the rate of return-to-work outcomes of individuals on an active workers’ compensation claim **(O)** (11) - Providing feedback and progress tracker by the digital scale to manage weight changes to other behaviors such as physical activity, diet, or sleep **(S)** (9) |
|  | **Clinical Process Improvement** | - Limited impacts on clinician workflow by the Precision Nudging intervention **(S)** (8) - Workload reduction of health professional **(O)** (6) - Removing additional steps for providers by the ability to capture patient data from medical records automatically **(S)** (8, 9) - The possibility of considering communication time and frequency according to provider capacity by removing additional steps **(S)** (8) - Maximizing the throughput of mammography schedules without creating additional stress on providers with the possibility of monitoring and adjustment of mammography schedules by CALO Mama Plus **(O)** (8) - Easy dissemination of CALO mama Plus in healthcare settings, due to its freely downloaded **(O)** (5) |
|  | **Cost Effectiveness** | - Mitigating the excess demand for screening centers by using this AI-based intervention **(O)** (8) - Cost saving for the health providers **(O)**(6) - Providing low-cost interventions for active workers’ compensation claims by AI **(O)** (11) - Cost-sensitive algorithms in OnTrack can predict human behavior **(S)** (10) |
| **Suggestions** | **Further Investigation** | - Exploring the effect of embedded conversational agents on a broader range of indicators of behavioral and experiential engagement (12) (OW) - Capturing process outcomes in greater detail, to inform future improvements to program elements, such as the virtual health coach’s language style, variety of language, specific intervention content, and alternative modes of output (11) (OW) - Understanding causal effecte by accomplishing the randomized controlled trial or quasi-experimental implementation (8) (OS) - Investigating the economics of a behavioral intervention to increase mammography uptake in a health system (8) (OS) - Including a longitudinal design to capture long-term effects and general health outcomes to observe the lifestyle behaviors improvement over time (7, 13) (OW) - Examining the role of motivation and self-efficacy in web-based interventions to improve diabetes management (7) (OW) - Testing and validating AI algorithms in a clinical setting on a larger sample to demonstrate their effectiveness, safety, and impact on patient outcomes (14-16) (TW) - Conducting a micro-randomized trial to evaluate the effectiveness of lapse prediction and subsequent intervention (10) (OW) - Investigating the clinical and statistical impact of recoding false positives into true positives when the system is aiming to predict and prevent a proximal outcome in real-time (10) (TW) - Confirmatory trial could provide evidence to help disseminate and implement CALO Mama Plus to enhance individual health (5) (TW) |
|  | **Stakeholder Collaboration** | - Requiring the collaboration between different stakeholders, including AI experts, mental health professionals, researchers, and users or patients in the successful implementation of AI in mental health care (14) (TW) - Cocreating solutions with people with diabetes and health care professionals and further development of AI technology have great potential to improve diabetes management in a more engaging and personalized manner (7) (TS) - Considering incorporating this digital therapeutic solution in the healthcare providers’ usual care to facilitate positive outcomes for participants willing to stop smoking (4) (TS) - The collaboration between different stakeholders ensures that AI solutions are clinically relevant, user-friendly, and aligned with best practices in mental health treatment (14). (TW) |
|  | **End User Consideration** | - Designing AI systems features according to the users' needs, preferences, and experiences to increase their acceptance and engagement (7, 14) (OW) - Involving end-users and therapists in the design process and conducting usability tests to promote the effectiveness of AI-based interventions (14) (OW) - Considering effective interventions for people with high time preference rates who have difficulty sustaining healthy behaviors (1) (TS) - Requiring the process of person-to-person communication for health interventions (1) (TS) - Considering the needs of different age groups (ranging from the elderly who often have more difficulty accessing in-person health care services, to youth and young adults, who tend to be less adherent to traditional models of diabetes prevention and may selectively benefit from technology-based models) (17) (OW) - Designing a more complex, longer conversation that uses more aspects of a clinician-delivered ML conversation to invoke more contemplation in participants (3) (TW) - Improving acceptability, appropriateness, and satisfaction as key issues to be addressed in future implementation (2, 7) (OS) - Considering visual impairments, ethnicity, and socioeconomic status in designing digital applications for patients with diabetes (7) (TW) - Considering different preferences in terms of delivery mode among participants to improve survey response rates (7) (TW) - Reviewing the recipe content by diabetes experts to provide a reliable and healthy personalized diet (7) (OW) - Needing the careful analysis of the users’ engagement across time to extract any effective factors (4) (OW) - Knowing the intervention types that encourage people to engage in autonomous health activities (1) (TS) - Optimizing user engagement by customization to varied ethnicities and socioeconomic levels (17) (TS) - Constantly improving the virtual assistant’s capabilities according to users’ expectations to help users set realistic expectations about virtual assistants’ abilities and avoid disappointment (18) (OW) |
|  | **Technical Improvements** | - Paying attention to the virtual assistant’s “personality”-adding humor - to produce a more natural, and less robotic conversational style (18) (TW) - Needing further development to incorporate dynamic and real-time updates to adapt to the user’s changing needs and circumstances (13) (OW) - Using personalized technical assistance in the system to facilitate accessibility by users (6) (OS) - Facilitates continued compliance over time and use data to inform the timing and content of intervention delivery (10) (OW) - Using more algorithms and real-time data sources to adapt the timing, frequency, context, and content for each participant (16) and generate more effective responses and reflections(3) (TS) - Assessing the app’s performance in real-world scenarios (13) (OW) - Assessing the performance of the chatbot via various mechanisms of action to increase engagement (12) (OS) - Initiate regular conversations between users and virtual assistants (daily notifications and providing tips or asking supportive questions) (18) (OW) - Gathering more comprehensive feedback from users regarding their meal planning and preparation experiences, to provide a more comprehensive evaluation of the app’s usefulness, usability, and overall user satisfaction (13) (OS) - Using objective tracking and personalized feedback to promote greater awareness of one’s lifestyle behaviors (9) (OS) |
|  | **Resource Allocation** | - Allocating enough resources to support intensive, ongoing trials to address issues in a timely manner and enhance user experience and trust in the virtual health assistant (18) (OW) - Requiring substantial expertise, time, and financial resources in developing and implementing chatbots (12) (OW) |

Legend: BDHQ= Brief self-administered Diet History Questionnaire; HRS= Health Record System

1. Okaniwa F, Yoshida H. Evaluation of dietary management using artificial intelligence and human interventions: nonrandomized controlled trial. JMIR Formative Research. 2022;6(6):e30630.

2. Watanabe K, Okusa S, Sato M, Miura H, Morimoto M, Tsutsumi A. mHealth Intervention to Promote Physical Activity Among Employees Using a Deep Learning Model for Passive Monitoring of Depression and Anxiety: Single-Arm Feasibility Trial. JMIR Formative Research. 2023;7(1):e51334.

3. Brown A, Kumar AT, Melamed O, Ahmed I, Wang YH, Deza A, et al. A Motivational Interviewing Chatbot With Generative Reflections for Increasing Readiness to Quit Smoking: Iterative Development Study. JMIR Mental Health. 2023;10:e49132.

4. Carrasco-Hernandez L, Jódar-Sánchez F, Núñez-Benjumea F, Conde JM, González MM, Civit-Balcells A, et al. A mobile health solution complementing psychopharmacology-supported smoking cessation: randomized controlled trial. JMIR mHealth and uHealth. 2020;8(4):e17530.

5. Nakata Y, Sasai H, Gosho M, Kobayashi H, Shi Y, Ohigashi T, et al. A smartphone healthcare application, CALO mama plus, to promote weight loss: a randomized controlled trial. Nutrients. 2022;14(21):4608.

6. Olano-Espinosa E, Avila-Tomas JF, Minue-Lorenzo C, Matilla-Pardo B, Serrano MES, Martinez-Suberviola FJ, et al. Effectiveness of a conversational chatbot (Dejal@ bot) for the adult population to quit smoking: pragmatic, multicenter, controlled, randomized clinical trial in primary care. JMIR mHealth and uHealth. 2022;10(6):e34273.

7. Bul K, Holliday N, Bhuiyan MRA, Clark CC, Allen J, Wark PA. Usability and Preliminary Efficacy of an Artificial Intelligence–Driven Platform Supporting Dietary Management in Diabetes: Mixed Methods Study. JMIR Human Factors. 2023;10:e43959.

8. Bucher A, Blazek ES, West AB. Feasibility of a Reinforcement Learning–Enabled Digital Health Intervention to Promote Mammograms: Retrospective, Single-Arm, Observational Study. JMIR Formative Research. 2022;6(11):e42343.

9. Graham SA, Pitter V, Hori JH, Stein N, Branch OH. Weight loss in a digital app-based diabetes prevention program powered by artificial intelligence. Digital Health. 2022;8:20552076221130619.

10. Forman EM, Goldstein SP, Zhang F, Evans BC, Manasse SM, Butryn ML, et al. OnTrack: development and feasibility of a smartphone app designed to predict and prevent dietary lapses. Translational behavioral medicine. 2019;9(2):236-45.

11. Brinsley J, Singh B, Maher CA. A Digital Lifestyle Program for Psychological Distress, Wellbeing and Return-to-Work: A Proof-of-Concept Study. Archives of Physical Medicine and Rehabilitation. 2023;104(11):1903-12.

12. Perski O, Crane D, Beard E, Brown J. Does the addition of a supportive chatbot promote user engagement with a smoking cessation app? An experimental study. Digital health. 2019;5:2055207619880676.

13. Amiri M, Li J, Hasan W. Personalized Flexible Meal Planning for Individuals With Diet-Related Health Concerns: System Design and Feasibility Validation Study. JMIR Formative Research. 2023;7:e46434.

14. Danieli M, Ciulli T, Mousavi SM, Riccardi G. A conversational artificial intelligence agent for a mental health care app: Evaluation study of its participatory design. JMIR Formative Research. 2021;5(12):e30053.

15. Hassoon A, Baig Y, Naiman DQ, Celentano DD, Lansey D, Stearns V, et al. Randomized trial of two artificial intelligence coaching interventions to increase physical activity in cancer survivors. npj Digital Medicine. 2021;4(1):168.

16. To QG, Green C, Vandelanotte C. Feasibility, Usability, and Effectiveness of a Machine Learning–Based Physical Activity Chatbot: Quasi-Experimental Study. JMIR mHealth and uHealth. 2021;9(11):e28577.

17. Zahedani AD, McLaughlin T, Veluvali A, Aghaeepour N, Hosseinian A, Agarwal S, et al. Digital health application integrating wearable data and behavioral patterns improves metabolic health. NPJ Digital Medicine. 2023;6(1):216.

18. Maher CA, Davis CR, Curtis RG, Short CE, Murphy KJ. A physical activity and diet program delivered by artificially intelligent virtual health coach: proof-of-concept study. JMIR mHealth and uHealth. 2020;8(7):e17558.
